# A New Reliable Performance Analysis Template for Quantifying Action Variables in Elite Men’s Wheelchair Basketball

**DOI:** 10.3389/fpsyg.2019.00016

**Published:** 2019-01-23

**Authors:** John Francis, Alun Owen, Derek M. Peters

**Affiliations:** ^1^School of Sport and Exercise Science, University of Worcester, Worcester, United Kingdom; ^2^Faculty of Engineering, Environment and Computing, Coventry University, Coventry, United Kingdom; ^3^School of Allied Health & Community, University of Worcester, Worcester, United Kingdom; ^4^Faculty of Health and Sport Sciences, University of Agder, Kristiansand, Norway

**Keywords:** sport performance analysis, Paralympic, reliability, validity, elite sport

## Abstract

This study aimed to develop a valid and reliable performance analysis template for quantifying team action variables in elite men’s wheelchair basketball. First action variables and operational definitions were identified by the authors and verified by an expert panel of wheelchair basketball coaching staff in order to establish expert validity. A total of 109 action variable were then placed into 17 agreed Categorical Predictor Variable categories. The action variables were then used to develop a computerized performance analysis template for post-event analysis. Each possession (*n* = 200) from an international men’s wheelchair basketball game was analyzed by the first author on two occasions for assessment of intra-observer reliability and by a coach and a performance analyst for inter-observer reliability. Percentage error and Weighted Kappa coefficients were calculated to compare the levels of error and agreement for each action variable. Intra-observer reliability demonstrated perfect or almost perfect agreement (<K0.980) and low percentage error values (<1.50%) for the 109 action variables within the 17 categories. Inter-observer reliability demonstrated perfect or almost perfect agreement (<K0.974) and low percentage error values (<3.00%) for the 109 action variables within the 17 categories. The template should be used in future for obtaining valid and reliable data in elite men’s wheelchair basketball.

## Introduction

Performance analysis aims to assist the decision making and learning of athletes, coaches and support staff (Sampaio et al., 2013; Hughes and Bartlett, 2015). Objective performance data are collected regarding the key actions and behavioral aspects of an individual’s and/or team’s performance (Sampaio et al., 2013) through specifically designed performance analysis templates and systems. The data are then utilized to provide feedback. Central to the quality of the feedback is the analyst’s ability to design an appropriate data collection template that will permit the collection of valid and reliable performance data.

If a sport performance analysis template can record a sports performance using precise definitions of actions and events and consistently produce similar or identical results each time it is used, it can be deemed both valid and reliable. However, previous performance analysis research has highlighted problems in the processes often undertaken to identify valid action variables and to develop a reliable performance analysis template (Watson et al., 2017; Jayal et al., 2018). Particularly in relation to the validity of defining action variables, performance indicators, operational definitions, and the reliability test procedures themselves (James et al., 2005; Hughes et al., 2012; Thomson et al., 2013; Hughes, 2015).

Hughes (2015) argued the presentation of reliability and validation procedures has increased immensely since Hughes et al. (2002) previously highlighted the need for the reliability of performance analysis templates to be clearly established within all studies. Prior to the 2007 special edition of the International Journal of Performance Analysis in Sport that focused on reliability issues in performance analysis, of the 77 empirical studies published, only 56% of the journal’s articles reported reliability procedures and only 42% included details detailing the validation procedures.

Within the special edition’s editorial, O’Donoghue (2007, p. i) stated the discipline “takes reliability very seriously because many methods involve human operators where there are many sources of measurement error.” Subsequently, the number of articles within the journal presenting information regarding the reliability procedures increased to 68% but the number of studies outlining the validation processes reduced to 40% (312 out of 462 articles that included empirical data between 2007 and 2015). Despite these clear recommendations, the importance of establishing and presenting both the validity and reliability of performance analysis templates is too often still overlooked. More recently, Watson et al. (2017) have reiterated this point and attempted to address the issue regarding validity and reliability of key performance indicators that discriminate between successful and unsuccessful rugby union teams. However, the issue of the collection of valid and reliable performance analysis data in less studied sports, e.g., wheelchair basketball, are no exception to this trend.

Wheelchair basketball is played by people with varying physical disabilities with a primary objective of scoring more baskets than their opponents (Frogley, 2010). To achieve this objective, the offensive team endeavors to progress the ball toward the basket by coordinating actions in an attempt to position themselves close to the basket, whilst the defensive team attempts to coordinate actions to restrict the offensive players’ space to shot and regain possession. The two teams consist of players with a range of disabilities, including amputations, birth defects, cerebral palsy, paralysis due to an accident and, spina bifida, and who are unable to play the running form of basketball (Gil-Agudo et al., 2010). The growth in the sport, now being played by over 105 nations (International Wheelchair Basketball Federation, 2016), has led to the performance gap between participation and qualification into a World Championships or Paralympic Games becoming increasingly difficult. Nations have had to become more tactically and technically strategic in the way athletes and teams prepare for competitions through turning to performance analysis (de Bosscher et al., 2008). The discipline, therefore, seems to be an excellent approach for increasing the technical and tactical understanding of wheelchair basketball demands, assisting coaches, athletes, classifiers and analysts with the ability to apply the findings in order to improve training plans and game management.

Each of the seven post-event wheelchair basketball performance analysis articles published, however, that have attempted to explore the technical and tactical demands of the sport using a form of performance analysis template (Vanlandewijck et al., 1995, 2003, 2004; Molik et al., 2009; Skucas et al., 2009; Gómez et al., 2014, 2015), have inherently questionable validity and reliability. These studies have relied on box score data, with no consideration of its validity or reliability and the (modified) comprehensive basketball grading system (CBGS) to provide an “objective” means of evaluating individual player performance. The CBGS was originally developed for use in running basketball and from a very small sample of games at a specific level of competition (Mullens, 1978), making it invalid for use in the wheelchair game. The CBGS records the frequency counts of shots, rebounds, and fouls drawing a game, concluding that the classification system proportionally represents the functional potential of the players. However, these findings offer limited tactical and technical insights into the key determinants of success and thus provide limited contextually rich data that can be used by coaches, players and staff to inform future practice. Furthermore, the post-event analysis completed in these studies and largely in performance analysis research differs from applied practice whereby the immediateness of the obtained results is of priority. Post-event analysis, however, allows for greater in-depth analysis and warrants a higher precision of accuracy due to the possibility that errors can be rectified (Arriaza and Zuniga, 2016).

Researchers have attempted to include wheelchair basketball specific variables in the modified-CBGS (Byrnes and Hedrick, 1994), however, the sport-specific variables were removed due to definitional errors identified as a result of the operators’ experience (Vanlandewijck et al., 2003, 2004). The CBGS and modified-CBGS data were also found to be highly correlated with one another. Reliability of these studies was assessed by inter-observer reliability procedures using a Pearson’s R Correlation, which has been criticized due to presenting miss-leading results as it is an assessment of relationship, not agreement (Liu et al., 2016). Despite this, researchers have elected to use this “evidence” to determine the quality of players’ games and made comparisons between functional classification groups, identifying that higher classified players achieved higher CBGS scores.

Furthermore, researchers have also claimed the findings from individual box score data can be used to provide an insight into team performance. Neither version of the CBGS, however, capture contextual and situational relevant data regarding team performance. Araújo and Davids (2016) argued that it is important to consider the interactive behaviors of players over time and recording these on a continuous or sequential basis. Researchers have identified the performance relationship between game status (e.g., Sampaio et al., 2010), line-up rotations (e.g., Clay and Clay, 2014) and the offensive-defensive dyads involved in sports (García et al., 2013), and thus by capturing this data it may be possible to provide meaningful objective augmented feedback (Araújo, 2017; Jayal et al., 2018). Passos (2008) also argued that the collection of discrete variables, as is the case with the (modified) CBGS, does not provide a true insight into an entire performance. Additionally, the seven studies did not mention how the action variables were established. Therefore, if the process of establishing the action variables is not outlined and the secondary box score data has been shown to be potentially incorrect, the data collected should not be used by coaches, players and support staff to inform decisions regarding team aspects of performance (Ziv et al., 2010). The (modified) CBGS is not suitable for measuring team performance in elite men’s wheelchair basketball.

Considering the above concerns within the discipline and specifically in wheelchair basketball regarding reliability, there is a need for a new post-event valid and reliable sports performance analysis template to assess a team’s performance in wheelchair basketball. The template is required to correctly identify and record the actions that occur during a game in a consistent manner, thus providing coaches, players and support staff with meaningful performance data to inform future decision making following games. The variables that are analyzed in the study can contribute to the players’ learning, thus increasing the likelihood of wheelchair basketball teams achieving performance success. As such, an adequate methodological process for quantifying action variables in elite men’s wheelchair basketball was required. Therefore, the aims of this paper were to (i) develop a valid performance analysis template in elite men’s wheelchair basketball and (ii) assess its intra-observer and inter-observer reliability by the lead author, a wheelchair basketball coach and a performance analyst intern.

## Materials and Methods

**FIGURE 1:**
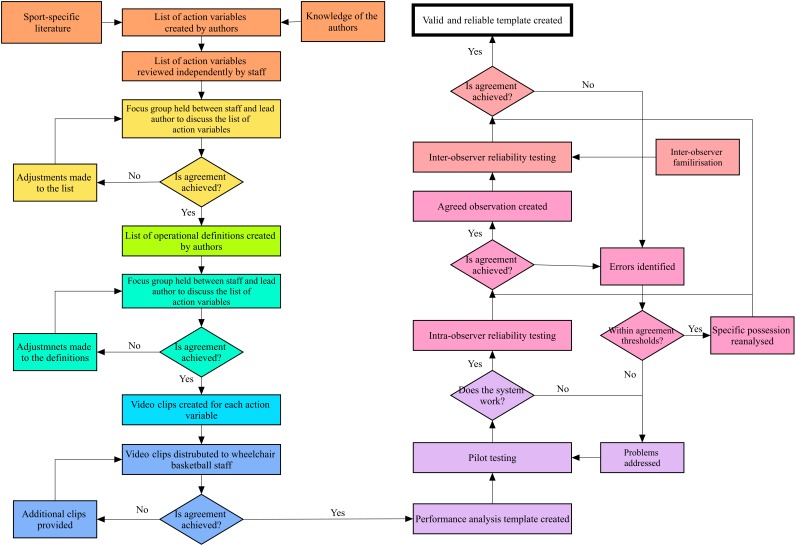
Diagram showing the systematic research process for developing a new performance analysis template [adapted from James et al. (2005) and Thomson et al. (2013)].

Following ethical approval from the University of Worcester’s Ethics and Research Governance Committee, the methodological approaches used by James et al. (2005) and Thomson et al. (2013) were followed as an initial framework. The framework was adapted and followed nine distinct stages; stages one to six relating to the validation process, stage seven developing the performance analysis template and stages eight and nine referred to establishing reliability (Figure 1).

### Validation Process

First, a list of 120 action variables was developed from previous wheelchair basketball literature and the knowledge of the authors. The action variables were initially grouped into 16 categories depending on the sub-phases that would occur during a single possession in the game. The action variables within each category were an exhaustive list of all behaviors that could occur which help toward understanding the sequential nature of a possession that would contribute toward scoring a basket.

Second, on receipt of written informed consent, developed in line with The British Association of Sport and Exercise Sciences code of conduct, from four elite wheelchair basketball staff, the list was circulated and the participants were given 1 week to scrutinize the information. The four staff members consisted of three elite wheelchair basketball coaches (Coach one: 20 years’ experience; Coach two: 19 years’ experience; Coach three: 19 years’ experience) and a member of support staff from an elite wheelchair basketball team (3 years’ experience). During the week, the staff were asked to review the list and provide their opinions as to whether the variables and categories would allow the collection of objective data regarding the sequential nature of a possession. The staff made notes on the list and returned it.

Third, adaptations were made to the action variables and categories during a focus group with all four staff and the lead researcher. Following the discussion, the adapted list comprised of 109 action variables placed into 17 categories. The Offense – End and Defense – End categories were combined into the End of Possession category, removing 18 action variables, the action variables within the Offense – Shot category were split into three categories (Shot Taken, Shot Point, and Shot Outcome) adding two action variables and the Defensive System category added five action variables to provide additional context to the possession.

Fourth, operational definitions were developed for each of the 109 variables using various resources (Frogley, 2010; Federation International Basketball Association, 2014; International Wheelchair Basketball Federation, 2014). The list of action variables and operational definitions was then re-circulated to each of the wheelchair basketball staff members who were given another week to comment.

Fifth, the staff identified any suggested amendments to the definitions during a second focus group. The definitions for “Zone” and “Highline” Defensive System were discussed and amended to add further clarity.

Sixth, video clips with overlaying text were created illustrating each action variable. The clips were circulated to the wheelchair basketball staff using external hard drives. Each member was given 1 week to watch the clips and ensure the overlaying text represented the operational definitions for each action variable. One staff member requested a further clip to illustrate the different types of Defensive System when a team were playing a “Highline” defense. The clip was circulated to all staff members. After watching the additional clip, the staff members confirmed the second video clip represented the overlaying text more accurately. No additional clips or amendments to the operational definitions were required, resulting in the final list of 109 action variables placed into 17 categories (Table 1).

**Table 1:** Operational definitions for the action variables in each category.

Category	Action variable	Definition
Quarter	Q1 Q2 Q3 Q4	A possession which occurs during the stated quarter of the game. The time in the game is indicated on the scoreboard. Each quarter lasts 10 min, with the clock stopping when the ball is dead (out of bounds, foul or the referee stops play).
	Over time	Once all four quarters have been played, a 5 min period of overtime will be played if the teams are drawing.
Game status	Winning	At the start of a possession, the team with the ball are currently leading on the scoreboard.
	Drawing	At the start of a possession, the team with the ball are currently drawing on the scoreboard.
	Losing	At the start of a possession, the team with the ball are currently losing on the scoreboard.
Home team Away team	The vest numbers of the on-court players, ranging from 0 to 99. For every possession, there will be five “Home Team” numbers and five “Away Team” numbers.	
Home classification Away classification	The classification of the on-court players according to the International Wheelchair Basketball Federation classification system (International Wheelchair Basketball Federation, 2016). For every possession, there will be five “Home Classification” numbers and five “Away Classification” numbers.	
Start of possession	Inbound – baseline	The referee will take the ball to either side of the backboard in the defensive half of the court where the play will begin. One player on the offensive team will push out of bounds behind the baseline and is given 5 s to pass the ball to a teammate.
	Inbound – endline	The referee will take the ball to either side of the backboard in the offensive half of the court where the play will begin. One player on the offensive team will push out of bounds behind the baseline and is given 5 s to pass the ball to a teammate.
	Sideline – front	The referee will take the ball to the location near the half-court line where the play will begin. One player on the offensive team will push out of bounds behind the sideline and is given 5 s to pass the ball to a teammate from within the offensive half of the court.
	Sideline – back	The referee will take the ball to the location near the half-court line where the play will begin. One player on the offensive team will push out of bounds behind the sideline and is given 5 s to pass the ball to a teammate from within the defensive half of the court.
	Defensive rebound	The defensive team gains possession of the ball after a missed shot that is not gathered by an offensive player.
	Offensive rebound	Possession starts when the offensive team retains possession of the ball after a missed shot.
	Free throw	An unopposed shot behind a line 15 feet from the basket, typically awarded to an offensive player who is fouled while in the act of shooting. Each free throw made is worth one point. A free throw is also known as a “foul shot.”
	Other start	Any other possession start, e.g., start of the game.
	Turnover	A turnover occurs when the offensive team loses possession of the ball to the opposing team, resulting from a handling error.
Shot taken	Shot	During the possession, the ball is propelled in an upward direction toward the basket in an attempt to score.
	No shot	During the possession, the ball is not propelled toward the basket or if the ball is propelled toward the basket when the shot clock is past 0.1 s resulting in a Violation Against.
Shot point	One	Following the awarding of a free-throw attempt, the ball is propelled toward the basket from the free-throw line.
	Two	The ball is propelled toward the basket from inside the three-point zone and the referee will raise one hand in the arm and holds up two fingers.
	Three	The ball is propelled toward the basket from outside the three-point zone and the referee will raise one hand in the arm and hold up three fingers.
	No shot	During the possession, the ball is not propelled toward the basket or if the ball is propelled toward the basket when the shot clock is past 0.1 s resulting in a Violation Against.
Shot outcome	Successful	A shot that falls through the ring and is awarded the relevant points by the referee, indicated by the number of fingers held up by his/her hand.
	Unsuccessful	A shot that does not fall through the ring and is rebounded by a player or a player is stopped due to a foul/violation or the ball goes out of bounds.
	No shot	During the possession, the ball is not propelled toward the basket or if the ball is propelled toward the basket when the shot clock is past 0.1 s resulting in a Violation Against
Shot clock remaining	6–0.1 S 12–7 s 17–13 s 24–18 s	The time remaining on the shot clock when the offensive player propels the ball toward the basket. The time is recorded when the ball is released from the shooting player’s hands and not when the ball hits the ring, backboard or when the basket is scored. 17–13 s is also triggered when a player’s free-throw attempt (successful or unsuccessful) would result in the shot clock counting down from 14 s.
	Dead	The time on the shot clock is stopped. This only happens when an unsuccessful free-throw attempt results in an additional attempt.
	No shot	During the possession, the ball is not propelled toward the basket or if the ball is propelled toward the basket when the shot clock is past 0.1 s resulting in a Violation Against.
Shot location	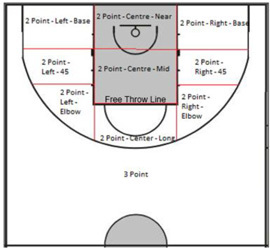	The location on the court where the shot attempt is taken from, this is measured from the position of the wheelchair’s front castors. When a Free Throw attempt is taken this is from the Free Throw Line.
	No shot	During the possession, the ball is not propelled toward the basket or if the ball is propelled toward the basket when the shot clock is past 0.1 s resulting in a Violation Against.
Man out offence	Equal numbers	The number of offensive and defensive players in the front-court is equal.
	Numbers advantage	The number of offensive players is different from the number of defensive players in the front-court.
End of possession	Foul against	The referee penalizes the team with the ball for infringing the rules of the game, resulting in a loss of possession.
	Foul for	The referee penalizing a player from the team without possession of the ball for infringing the rules of the game.
	Violation against	The referee awards the defensive team with a throw-in at the place nearest to the infraction of the rules.
	Defensive rebound	The defensive team secure possession from an unsuccessful shot.
	Offensive rebound	The offensive team maintains possession from an unsuccessful shot.
	Basket scored	The referee awards the offensive team with either a one-point, two-point or three-point score dependent on the location and circumstance of the shot.
	Other	The possession ends by another means, e.g., referee stopping play due to a player out of their wheelchair.
	Out of bounds	The ball goes crosses the field of play and results in the offensive team losing possession.
	Free throw	The referee awards a player with an unhindered shot in basketball made from behind a set line due to being fouled by an opponent.
	Handling error	A player from the offensive team loses possession through a backcourt violation, traveling or the opposition stealing the ball.
Defensive system	1 Man press 2 Man press 3 Man press 4 Man press 5 Man press	The stated number of defensive players applying pressure in the backcourt at the point when the ball is inbounded.
	Highline	The defensive players initially set up above the free throw line in a straight line between each sideline and force offensive players toward the sideline.
	Zone	The defensive players initially drop back to around the key before either staying put or pushing out toward the three-point line.
	No defensive system	The defensive players are unable to adopt a system as the offensive team attack the basket too quickly, e.g., from a turnover or the defensive system adopted when a player is taking a free-throw attempt.
Defensive outcome	Successful defense	The defensive team stop the offensive team from scoring and secure possession. If the team stop the offensive team from scoring but fail to secure possession the Defensive Outcome is Unsuccessful.
	Unsuccessful defense	The defensive team are unable to stop the offensive team from scoring or from re-securing possession.
Possession	Maintained	The offensive team re-secure possession.
	Lost	The defensive team are unable to secure possession.
	Basket scored	The offensive team score a basket.

### Template Development

**FIGURE 2:**
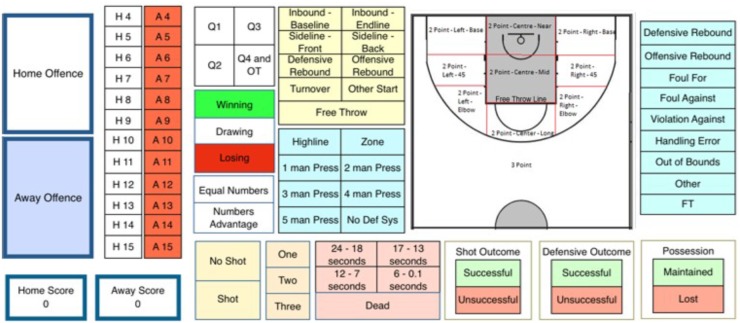
Team performance analysis template for coding wheelchair basketball performance.

Following the validation process (stages one to six above); a performance analysis template was created in SportsCode Elite Version 10 during stage seven by the lead researcher, the four wheelchair basketball staff and the performance analysis intern. The template underwent two pilot tests on a randomly selected elite wheelchair basketball game from a pre-tournament held in 2015. As a result of this pilot, the buttons were resized and positioned in their category group (Figure 2).

### Reliability Process

#### Intra-Observer Reliability Assessment

During stage eight, one game of elite male international wheelchair basketball was selected at random from the 2015 European Wheelchair Basketball Championships. The footage was imported into SportsCode Elite Version 10 and converted into a “SportsCode Project” analyzed post-game and viewed at normal playback speed (25 keyframes per second). If necessary, the playback speed was adjusted to ensure events were observed and recorded accurately. Multiple actions within a category could be recorded. For example, if the player was fouled in the act of scoring a successful basket then the End of Possession category would automatically record “Basket Scored” and “Foul For.” In addition, the home and away team numbers were checked against the official tournament website and the players’ classifications verified on the International Wheelchair Basketball Federation’s player database.

Levels of agreement with Weighted Kappa coefficients (Cohen, 1968) and percentage error values (Bland and Altman, 1999) were calculated for each category. The interpretation of Weighted Kappa coefficients within the field of performance analysis has been demonstrated by Lamas et al. (2015); with the following values being utilized: “<0 less than the chance agreement, 0.01–0.20 slight agreement, 0.21–0.40 fair agreement, 0.41–0.60 moderate agreement, 0.61–0.80 substantial agreement, and 0.81–0.99 almost perfect agreement” (Landis and Koch, 1977, p. 165). Whilst, the level of reliability for each category when using the percentage error value was deemed acceptable when less than five per cent error was identified (Hughes et al., 2002).

For intra-observer procedures, 100 Home Offense and 100 Away Offense possessions were analyzed on two occasions with a period of 4 weeks between the two observations. The two observations were exported as categorical variables from SportsCode using the “Sorter” function into Microsoft Excel. The 400 rows of data were transferred into a CSV file (Supplementary Data Sheet S1) and imported into R (R Core Team, 2015). Weight Kappa coefficients and percentage error values were calculated for each category to determine intra-observer agreement levels between the two observations. Where categories did not demonstrate perfect agreement or establish a zero per cent error, the source of the discrepancy was identified and the specific possession was re-observed to create an agreed observation.

### Inter-Observer Reliability Assessment

Following the establishment of an agreed observation, stage nine involved a wheelchair basketball coach and a performance analysis intern completing an observation of the same game, enabling the completion of an inter-observer reliability test. The wheelchair basketball coach, who had 19 years of sport-specific experience, was involved in the classification of action variables and had a year of experience using a similar performance analysis software program (Dartfish TeamPro, Switzerland). The performance analysis intern had 9 months experience of performance analysis in wheelchair basketball) and 3 years of experience as a performance analyst in rugby union using SportsCode Elite.

The coach and performance analyst intern accessed the action variables, operational definitions and the accompanying video clips 2 weeks prior to conducting the observations to help familiarize themselves with the specific behaviors they were required to record. In addition, the coach and the intern were allowed to code a pre-tournament game between the two competing nations to assist with learning the performance analysis template and the software. Familiarization varied in time for the two operators, with the coach completing four sessions of 2 h over a 5 day period and the intern undertaking an additional 2-h session before both individuals felt they were able to complete the reliability test (O’Donoghue, 2014). The testing was conducted 1 day after they had completed their final familiarization session. The coach and the intern focused on observing the entire game, which equated to 200 possessions. Weighted Kappa coefficients and percentage error values were calculated for each category to determine inter-observer agreement levels with the agreed observation being first compared against the coach’s observation and second against the performance analyst intern’s observation. Finally, the coach’s, performance analyst intern’s and the agreed observation were triangulated and expressed as Weighted Kappa coefficients and percentage error values.

## Results

### Intra-Observer Reliability Test

Cohen’s Weighted Kappa demonstrated perfect agreement (K1.000) for 12 categories and almost perfect agreement (K0.987–0.994) for the remaining five categories between the first (Ob1) and second observation (Ob2) (Table 2). Percentage error reported zero error for the same 12 categories and below the five per cent acceptable error percentage for the remaining five categories.

**Table 2:** Intra-observer agreement reported using Cohen’s Weighted Kappa (K) and percentage error between the first observation (Ob1) and the second observation (Ob2).

	Quarter	Home team	Home classification	Away team	Away classification	Game status	Start of possession	Man out offence	Shot taken	Shot point	Shot outcome	Shot location	Shot clock remaining	End of possession	Defensive system	Defensive outcome	Possession
Ob1 vs.	K1.000	K1.000	K1.000	K1.000	K1.000	K1.000	K0.981	K1.000	K1.000	K1.000	K1.000	K0.988	K0.980	K0.981	K0.980	K1.000	K1.000
Ob2	0.00%	0.00%	0.00%	0.00%	0.00%	0.00%	1.50%	0.00%	0.00%	0.00%	0.00%	1.00%	1.50%	1.50%	1.50%	0.00%	0.00%

### Inter-Observer Reliability

#### Agreed Observation Versus Coach’s Observation

The test demonstrated perfect agreement (K1.000) and zero percentage error for ten categories and almost perfect agreement (K0.974–0.993) and within the acceptable percentage error threshold (0.50–1.50%) for the remaining seven categories (Table 3). The Man-Out Offense category recorded the lowest Weighted Kappa coefficient (K0.974) but almost a zero percentage error value (0.50%). By comparing the frequency counts for each action variable between the two observations within this category, it was identified that no action was recorded for one possession by the coach resulting in the discrepancy.

**Table 3:** Inter-observer agreement reported using Cohen’s Weighted Kappa (K) and percentage error between the agreed observation (Ob3), the coach’s observation (Ob4), and the performance analyst intern’s observation (Ob5).

	Quarter	Home team	Home classification	Away team	Away classification	Game status	Start of possession	Man out offence	Shot taken	Shot point	Shot outcome	Shot location	Shot clock remaining	End of possession	Defensive system	Defensive outcome	Possession
Ob3	K0.993	K1.000	K1.000	K1.000	K1.000	K1.000	K0.981	K0.974	K1.000	K0.992	K1.000	K0.994	K0.981	K1.000	K0.993	K1.000	K1.000
vs. Ob4	0.50%	0.00%	0.00%	0.00%	0.00%	0.00%	1.50%	0.50%	0.00%	0.50%	0.00%	0.50%	1.50%	0.00%	0.50%	0.00%	0.00%
Ob3	K1.000	K1.000	K1.000	K1.000	K1.000	K1.000	K0.994	K1.000	K1.000	K1.000	K1.000	K0.983	K0.981	K0.987	K0.993	K1.000	K1.000
vs. Ob5	0.00%	0.00%	0.00%	0.00%	0.00%	0.00%	0.50%	0.00%	0.00%	0.00%	0.00%	1.50%	1.50%	1.00%	0.50%	0.00%	0.00%
Ob3 vs. Ob4 vs.	K0.996	K1.000	K1.000	K1.000	K1.000	K1.000	K0.987	K0.983	K1.000	K0.995	K1.000	K0.988	K0.974	K0.991	K0.991	K1.000	K1.000
Ob5	0.50%	0.00%	0.00%	0.00%	0.00%	0.00%	1.50%	0.50%	0.00%	0.50%	0.00%	1.50%	3.00%	1.00%	1.00%	0.00%	0.00%

#### Agreed Observation Versus Performance Analyst Intern’s Observation

The test demonstrated perfect agreement (K1.00) and zero percentage error with 12 categories and almost perfect agreement (K0.981–0.993) and within the five per cent error limit (0.50–1.50%) with five categories (Table 3). The Shot Clock Remaining category recorded the lowest Weighted Kappa coefficient (K0.981) and highest error percentage (1.50%) as a result of three disagreements.

#### Triangulation of Coach’s, Performance Analyst Intern’s, and Agreed Observation

Through reporting the Weighted Kappa coefficients and percentage error values of the 17 categories, 9 categories demonstrated perfect agreement and zero percentage error, and 8 categories produced almost perfect agreement (K0.974–0.996) and within the five per cent error threshold (0.50–3.00%). Three categories, Shot Location, Start of Possession and Shot Clock Remaining, reported the largest number of discrepancies amongst the variables within each action variable (Table 3). The triangulation results for the Shot Location category highlight the category is the most susceptible to producing errors, however, the Weighted Kappa coefficient and percentage error values are still within the acceptable thresholds for agreement levels.

## Discussion

This paper set out to develop a unique valid and reliable performance analysis template for wheelchair basketball. To achieve this aim, the methodological procedures to develop a template completed by James et al. (2005) and Thomson et al. (2013) were adapted. This involved completing a nine-stage methodological process, which included a validation process, template development and reliability assessment. To address the limitations of the (modified) CBGS, it was necessary to employ the knowledge of sport-specific staff to assist in identifying contextually relevant action variables as well as drawing on the existing sport-specific literature. The four members of staff that were used in the paper provided a qualitative contribution through focus groups to further enhance the final list of 109 action variables and operational definitions.

The template was developed to be used post-event, with the ability to extract data as total frequency counts or as successive, discrete possessions. The development of the template built on Cooper et al. (2007) idea of dividing an observation into specific time cells. It also agreed with Thomson et al. (2013) work that this process was a sufficient method for assessing test-retest analysis. However, rather than dividing the observed performance into 2 min or 10-s time cells, each possession, which could last up to 24 s, was used. As outlined above, within each possession, irrelevant of the duration, each observer collected information pertaining to 17 categories.

Intra-observer and inter-observer reliability tests highlighted that the accuracy of all observations was excellent for the notation of all 109 action variables and 17 categories with inter-observer reliability slightly lower than intra-observer reliability. The coach’s observation achieved the lowest Weighted Kappa coefficient for the Shot Clock Remaining category whilst the performance analyst intern achieved the lowest Weighted Kappa coefficient for the Man-Out Offense category.

Previous research in boxing and rugby union have identified that it is not unexpected for the level of inter-observer reliability to be inferior to intra-observer reliability (Thomson et al., 2013: intra-observer agreement ranged from 80–100% whereas inter-observer agreement ranging from 33–100%; James et al., 2005: intra-observer agreement ranged from 1.97+3.14% whilst inter-observer agreement ranged from 11.09+8.61%), but all observations in this paper fell within the adequate levels of reliability. It is clear, however, that an adequate period of template piloting, familiarization and training was key to obtaining these excellent levels of reliability. The small disagreements identified between the observations could be due to the dynamic nature of the sport whereby observers are attempting to record action variables quickly and thus may incorrectly click on a closely related button rather than missing an action at all. Examples of this were identified when the coach coded the possession starting as an “Offensive Rebound” whereas the agreed observation coded the possession starting as a “Defensive Rebound.” It could also be argued that whilst operational definitions should be clear to distinguish between the two rebound types, they share a number of characteristics and thus may explain the disagreement.

The use of two reliability statistical approaches, Weighted Kappa coefficients (Cohen, 1968) and percentage error values (Bland and Altman, 1999), provided a useful cross-checking method for determining the reliability of the template. The concept of percentage error allowed directed comparisons of agreement to be made irrespective of the scaling between observers (Hopkins, 2000). Thus, it enabled the identification of errors and determined if these were random (McHugh, 2012). Whilst the Weighted Kappa tests acknowledged that in some instances no operator could be sure of the action to record (McHugh, 2012) and provided credit when two observers recorded adjacent values, for example, in the Shot Location category. The use of both percentage error and weighted kappa statistics to assess intra-observer and inter-observer is recommended in the development process of a performance analysis template.

It is important to note, however, that this template was developed for post-event analysis, and thus changes would be required if the goal was to use the template in real-time analysis. The action variables included within the template were carefully considered to ensure meaningful and contextually relevant information was captured. Additional action variables could be added to the template to assist in strengthening the profile of an elite team’s performance regarding different tactical approaches, however, this would likely increase the time taken to analyze the wheelchair basketball performance and interpret the data. Subsequently, if additional modifications were made to the action variables, operational definitions, categories, or template, further reliability assessment would be required. Nevertheless, the current template provides the grounding for future attempts to identify the key tactical determinants of team success in elite wheelchair basketball and the processes undertaken to produce the template provide a framework for the development of future templates in all team sports.

## Conclusion

The paper provides an improved methodological process to establish a valid and reliable performance analysis template, that in this article we have used to produce accurate and reliable observations of key performance behaviors in a sequential nature within elite male wheelchair basketball. Additionally, the template has enabled the collection of most actions that occur in a wheelchair basketball possession whilst also recording the actions of the opposition, allowing for a context-specific insight to be gained. The current template should now be used by wheelchair basketball coaches, analysts and researchers to collect valid and reliable performance data at zonal qualification tournaments, world championships, and Paralympic Games to help identify the key tactical determinants of team success and subsequently to underpin both performance enhancing training and within-game practices.

## Data Availability Statement

The datasets generated for this study can be found in the Worcester Research and Publications collection (https://eprints.worc.ac.uk/id/eprint/7334 and https://eprints.worc.ac.uk/id/eprint/7332).

## Author Contributions

JF devised the structure of the manuscript, collected and analyzed the data, and drafted the manuscript. AO provided the guidance and support for statistical analyses and reporting, and commented on the final draft. DP devised the structure of the manuscript, oversaw the whole research process, commented on drafts and approved the final draft.

## Conflict of Interest Statement

The authors declare that the research was conducted as part of a Ph.D. research project between the University of Worcester and British Wheelchair Basketball.

## Supplementary Material

DATA SHEET S1 Raw intra-observer and inter-observer reliability data.
